# Triple negative breast cancer: shedding light onto the role of pi3k/akt/mtor pathway

**DOI:** 10.18632/oncotarget.10858

**Published:** 2016-07-26

**Authors:** Daniela Massihnia, Antonio Galvano, Daniele Fanale, Alessandro Perez, Marta Castiglia, Lorena Incorvaia, Angela Listì, Sergio Rizzo, Giuseppe Cicero, Viviana Bazan, Sergio Castorina, Antonio Russo

**Affiliations:** ^1^ Department of Surgical, Oncological and Oral Sciences, Section of Medical Oncology, University of Palermo, Palermo, Italy; ^2^ Fondazione Mediterranea “G.B. Morgagni”, Catania, Italy; ^3^ Department of Biomedical and Biotechnological Sciences, University of Catania, Catania, Italy

**Keywords:** ER, HER2, PI3K/AKT/mTOR inhibitor, target therapy, triple negative breast cancer

## Abstract

Breast cancer is one of the most widespread carcinoma and one of the main causes of cancer-related death worldwide, especially in women aged between 35 and 75 years. Among the different subtypes, triple negative breast cancer (TNBC) is characterized by the total absence of the estrogen-receptor (ER) and progesteron-receptor (PR) expression as well as the lack of human epidermal growth factor receptor 2 (HER2) overexpression or gene amplification. These biological characteristics confer to TNBC a higher aggressiveness and relapse risk along with poorer prognosis compared to other subtypes. Indeed, 5-years survival rate is still low and almost all patients die, despite any adjuvant treatment which at moment represents the heading pharmacological approach. To date, several clinical trials have been designed to investigate the potential role of some molecular markers, such as VEGF, EGFR, Src and mTOR, for targeted treatments in TNBC. In fact, many inhibitors of the PI3K/AKT/mTOR pathway, frequently de-regulated in TNBC, are acquiring a growing interest and several inhibitors are in preclinical development or already in early phase clinical trials. In this Review, we investigated the role of the PI3K/AKT/mTOR pathway in TNBC patients, by summarizing the molecular features that led to the distinction of different histotypes of TNBC. Furthermore, we provided an overview of the inhibition mechanisms of the mTOR and PI3K/AKT signaling pathways, highlighting the importance of integrating biological and clinical data for the development of mTOR inhibitors in order to implement targeted therapies for TNBC patients.

## INTRODUCTION

Breast cancer (BC) is one of the most widespread carcinoma and one of the main causes of cancer-related death worldwide especially in women aged between 35 and 75 years [1]. In the last few years new molecular markers have been studied to provide new insights on BC heterogeneity but also to better understand and predict tumor behavior during treatment. It is now well established that BC can be classified into different groups according to gene expression profiles [2-5]. This new classification will certainly provide new insights into the BC biology and will probably drive treatment decisions in the near future by microarray analysis that soon will switch the clinical approach to different illnesses by giving a huge support to conventional pathology (morphology and immunohistochemistry) [6].

The different BC subgroups detected by their different gene expression profiling are below described as discussed in San Gallen Expert Consensus report:

Luminal A subgroup is characterized by estrogen/progesterone receptor (ER/PR) positivity, lower expression of Ki-67 (< 20%) and HER2 lack, accounting for the 50% of all invasive BCs; Luminal B subgroup results characterized by ER/PR positivity or variable expression of HER2 (+ or -), accounting for the 10-20% of all invasive BCs [7]; HER2 overexpression subtype is characterized by ER/PR negativity and HER2 strong positivity. This subtype accounts for 15% of all invasive breast cancer; Basal like breast cancer (BLBC) subtype exhibits an expression profile similar to that of the epithelial cell mammary tissue and includes triple negative breast cancer (TNBC) [8-10].

Among the different subgroups, TNBC presents biological characteristics that confer higher aggressiveness and relapse risk along with worse outcome in comparison to other subgroups. Several studies showed that PI3K/AKT/mTOR signaling is often altered in TNBC patients [11].

## SELECTION CRITERIA

We have searched trials using Medline (PUBMED), EMBASE and COCHRAINE database, the following search strategy (“TNBC” [MESH] AND (“PI3K” [tiab] OR “mTOR” [tiab]) AND (“ER” [tiab] OR “PgR” [tiab]) and free text terms as “PI3K” [tiab], “triple negative breast cancer” [tiab] and “mTOR” [tiab].

## TRIPLE NEGATIVE BREAST CANCER

TNBC is characterized by lack of ER and PR expression as well as the absence of HER2 [12, 13]. The percentage of new TNBC diagnosis is variable, but it mainly ranges between 9% and 16% with a higher frequency in young women carrying *BRCA1* gene mutation, showing a strong correlation with ethnic origin (in particular, African-American and Hispanic women) [14-17]. TNBC also shows greater size and tumor burden, and often is a more aggressive high grade tumor [18, 19].

TNBC patients show a higher susceptibility to develop metastases, resulting in an unfavorable clinical outcome compared to other subgroups [20-22].

Although TNBC patients initially respond to neoadjuvant treatments, only 30% of them will exhibit a survival higher than 5-years following the first diagnosis, reflecting the aggressiveness of this subtype [23, 24]. Patients with *BRCA1* mutation are often diagnosed with TNBC but not all TNBC are *BRCA1* positive. Nevertheless, it been shown that TNBC not carrying *BRCA1* mutation, behave similarly to *BRCA1*-deficient tumors, showing also similar gene expression profiles [25, 26].

The growing interest in the TNBC biology allowed to develop trials investigating new drug targeting potential biomarkers such as VEGF, EGFR, Src and mTOR [27]. Moreover, the introduction of tumor molecular features in the characterization of TNBC has led to a further subtype labeling. Indeed, 6 new TNBC subtypes have been identified [28]:

Basal-like 1 (BL1) and Basal-like 2 (BL2) subtypes: both are characterized by up-regulation of gene and cellular markers mainly implicated in cell growth. In fact, Ki-67 expression and nuclear fraction staining are higher (BL1+BL2 = 70%) if compared to other subtypes (42%). All these features combined together indicate that more efficient treatment for BL TNBCs could be that directed against the mitotic apparatus such as a taxane-based therapy [29-32]. Furthermore, BL2 shows the involvement of a different plethora of growth factors and receptors (EGF, EGFR, NGF, MET, Wnt/β-catenin, IGF1R and EPHA2).

Immunomodulatory subtype: it is characterized by immune system gene signature similar to that of medullar BC determining its better clinical outcome.

Mesenchymal and mesenchymal stem-like subtypes: both are characterized by increased expression of gene and cellular markers involved in cell motility (Rho pathway), extracellular matrix-receptor interaction and differentiation (Wnt/β-catenin, ALK, TGF-β pathways). The mesenchymal stem-like subtype shows reduced expression of proliferative genes and enrichment of genes involved in several signaling pathways, including the inositol phosphate-dependent signaling pathway, EGFR, PDGF, and ERK1/2 signaling. Moreover, notable is the contribute of the adipocytokine signaling and ABC transporter. Both subtypes exhibit gene expression pattern and chemoresistance similar to metaplastic BC [28].

Luminal androgen receptor (LAR) subtype: this TNBC subgroup is ER-negative and is characterized by high deregulation of hormone-dependent pathways. In particular, the androgen receptor pathway seems to play a pivotal role in inducing expression of specific genes of the LAR subtype [33-35].

Indeed, androgen receptor mRNA expression has been shown to be considerably increased (9-fold) with respect to the other subtypes. Furthermore, tumors here classified show the up-regulation of a plethora of downstream targets and co-activators of the androgen receptor signaling [36-38].

## TARGET THERAPY IN TNBC

The major issue for targeted therapy against TNBC is the lack of specific oncogene drivers due to wide BC heterogeneity [39-41].

To date, the main approach in TNBC treatment remains the chemotherapy, in particular the administration of anthracyclines, taxanes and/or platinum compounds able to target dividing cells. Unfortunately, not all chemotherapy treated patients show a favorable outcome and is still unclear whether treatment choices should be personalized among the different TNBC subtypes [42, 43].

Maybe a possible solution would be represented by the new proposed genetic signature tools as suggested by the recent MINDACT trial results, in the order to avoid the aggressive treatment to non-responder patients. MammaPrint genetic study allowed to identify a large group of patients which showed a good five-year progression-free survival (PFS) good though they have not received adjuvant treatment (AACR Annual Meeting 2016).

Indeed, for pre-operative treatment pCR (pathological Complete Response) would represent the best surrogate survival end-point for TNBC patients and it results doubled if platinum compounds are added to conventional therapy compared to the worse outcome achieved by TNBC patients showing residual disease [44, 45].

Given the aforementioned issues for management of TNBC patients, studies are urgently needed to improve the use of target therapies. The major difficulty is to discover actionable target because of wide heterogeneity of the disease. In fact, clinical trials on TNBCs that aim to point out a particular receptor fail to demonstrate an evident clinical benefit. One of the most important involved receptors is EGFR, that is upregulated in about 60% of TNBCs, whose trial investigating chemotherapy plus EGFR targeted agent *versus* chemotherapy alone showed a modest advantage in terms of response rate (RR) (33% vs 28%) [46]. Among the reasons why studies were not able to underline a significant clear advantage of these new proposed drugs, we should not take into account the heterogeneity of the disease that probably masks the real effect of the drug in a smaller population carrying the right target [47]. Recent studies are investigating a number of promising molecules and, thanks to some favourable hopeful results, a growing interest is developing about some specific signaling pathways such as PI3K/AKT/mTOR. [48-50].

### PI3K/AKT/mTOR signaling pathway

**Figure 1:**
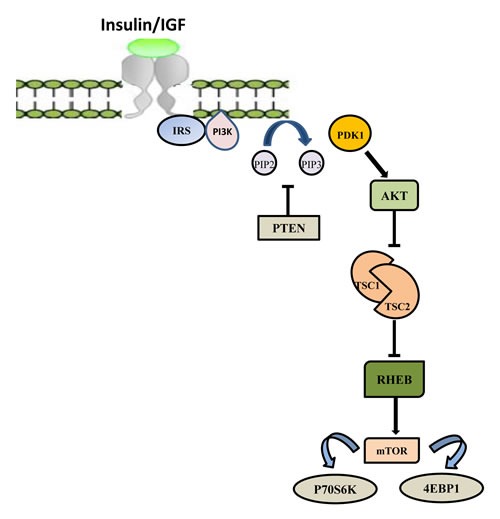
PI3K/AKT/mTOR signaling pathway The PI3K signaling pathway is triggered by activation of receptor tyrosine kinase (RTK) in cell membrane. After binding to the growth factors, the intracellular domain of RTK is phosphorylated, and PI3K is activated. Activated PI3K phosphorylates PIP2 to produce PIP3. The tumor suppressor phosphatase and tensin homolog (PTEN) could negatively regulate this process via dephosphorylation of PIP3. Activated PIP3 could prompt the phosphorylation of Akt and further stimulate the Akt-­mediated activation of downstream targets, including the Bcl-2 family members, Mdm2 and tuberous sclerosis complex 2 (TSC2). Activated Akt inhibits the Rheb GTPase activity of TSC1/2 complex by phosphorylating TSC2. Then, activated Rheb promotes mTOR complex 1 (mTORC1) to phosphorylate p70S6 and 4E binding protein1 (4EBP1), resulting in dysregulation of protein synthesis and cell survival.

PI3K/AKT/mTOR (PAM) represents the main signaling pathway responsible for cell proliferation, survival, metabolism and motility regulation and is often activated in BC [51-54] (Figure 1). A heterodimeric molecule belonging to the lipid kinases, phosphoinositide 3-kinase (PI3K), is the major component of this pathway. Based on structure, regulation mechanism and lipid substrate specificity, they can be categorized in three classes, but the class I PI3K is the more dysregulated in cancer [55].

PI3K signaling pathway starts following the binding of a growth factor or ligand to a variety of tyrosine kinase (TK) receptors, including HER proteins and IGF-1 receptors [56-58].

In its activated form PI3K phosphorylates phosphatidylinositol 4,5-bisphosphate (PIP2) to phosphatidylinositol 3,4,5-triphosphate (PIP3) which represents the docking site for AKT kinase. AKT activation leads to protein synthesis and cell growth by activating mTOR through TSC1/2 [59-61].

The main PI3K counteracting protein is the PTEN phosphatase, which acts by converting PIP3 to PIP2 [62]. Therefore, PIP3 results activated by PI3K and negatively controlled by PTEN [63].

Moreover, PIP3 levels seem to be also tightly modulated by another tumor suppressor, inositol polyphosphate 4-phosphatase type II (INPP4B), which dephosphorylates PIP3 to PIP2 [64].

Many research works report a higher incidence of *PTEN* and *PI3K* mutations in TNBC patients with respect to other histological subtypes [65].

A downstream component of PI3K/AKT pathway is mTOR which exists in two functionally different complexes, mTOR complex 1 (mTORC1) and mTOR complex 2 (mTORC2). mTORC1 is responsible for the activation of protein translation process by promoting mRNA translocation and is also involved in metabolism and lipid synthesis [66]. mTOR downstream substrate is S6K1 which in turn can phosphorylate estrogen determining its activation with a mechanism independent of the ligand [67-69].

On the other hand, mTOR complex 2 is involved in the organization of actin cytoskeleton and, at the same time, regulates AKT phosphorylation. The importance of mTOR complexes and their pathways is fundamental in clinics due to the ability of many drugs to target selectively mTORC1 [70]. Indeed, studies conducted on TNBC murine models highlighted the effects of the inhibitor Dactolisib in controlling the whole mTOR pathway [71]. The frequency of mTOR pathway activation is higher in TNBC compared to other subtypes and is often correlated with poor prognosis [22, 72, 73]. Moreover, the up-regulation of PAM signaling induces resistance to hormone treatment, HER2-targeted treatment and cytotoxic therapy [74].

## MAMMALIAN TARGET OF RAPAMYCIN INHIBITORS

Everolimus (RAD001) is a mTOR inhibitor exhibiting vast anticancer activity in preclinical studies [75]. The combined treatment of rapamycin with paclitaxel in cell lines altered in the PI3K/AKT/mTOR signaling has been shown to increase effectiveness of treatment in TNBC [76]. This rationale has been explored in different clinical experiences. In a phase II study, Meyer and collaborators investigated the addition of everolimus 5 mg/day for 12 weeks to a short course pre-operative chemotherapy regimen containing weekly cisplatin (25 mg/m²) + paclitaxel (80 mg/m²) in patients affected by stage II / III TNBC demonstrating that no improvement was detected in the pCR after surgery and RR, following the addition of RAD001 [77]. Another phase II randomized study aimed to investigate the addition of everolimus to paclitaxel in neoadjuvant sequential regimen containing anthracyclines. Fifty women affected by stage II/III TNBC were subjected to a therapy with paclitaxel 80 mg/mq for 12 weeks or paclitaxel 80 mg/mq + everolimus 30 mg/day orally for 12 weeks followed by an FEC scheme (5-FU 500 mg/mq, epirubicin 100 mg/mq and cyclophosphamide 500 mg/mq every 3 weeks for four cycles) [78]. The addition of everolimus, although well tolerated, did not add any significant benefit in terms of 12-week-RR (48% *versus* 30% in favour of everolimus) and pCR (30% *versus* 26% in favour of everolimus) [79].

For the same principles, everolimus was tested in combination with carboplatin. In particular, Singh et *al*. enrolled 25 patients affected by metastatic TNBC who underwent to a 3-weekly chemotherapy regimen containing carboplatin AUC6 (or decreased to AUC5/4) + everolimus 5 mg/day. The treatment has shown significant hematologic toxicity especially with regimens containing carboplatin AUC6/5, but was well tolerated with AUC4, demonstrating a clinical benefit rate > = 6 months of 28% with a total mOS of 16.6 months and mPFS of 3 months [80].

Despite TNBC is HER2- [81], RAD001 has also been tested in a regimen with anti-HER2 drugs since EGFR is overexpressed and upregulated in about 50% of TN tumors [82, 83], providing a strong rationale to investigate the association between an anti-EGFR and a mTOR inhibitor in order to overlap the resistance to anti-EGFR agents [84, 85]. Although the mTOR inhibitors paradoxically trigger the AKT pathway [86], this activation could probably serve as resistance mechanism to mTOR inhibitors thus explaining the poor performance of these drugs when used as a single agent [78, 87]. On this basis, Liu et *al*. in their experience have shown that the addition of everolimus could sensitize BC cells to anti-EGFR drugs (lapatinib) [88], demonstrating that this association may be responsible for an increased apoptosis in some TNBC cell lines and murine xenograft progression compared to the same drugs used in monotherapy [89]. The main clinical studies concerning the function of mTOR inhibitors in TNBC and currently under evaluation are reported in Table 1.

In another important work, Zhang et *al*. created a panel of seven patient-derived orthotopic xenografts from primary and metastatic neoplastic tissue having histological and immunohistochemical features matched between patient and their corresponding xenografts. Neoplasms were divided on the basis of the above characteristics in different TNBC subtypes and the authors created a response signature to mTOR inhibitors demonstrating that BLBC also possessed the highest expression rate of the genes belonging to the PI3K/AKT pathway and the highest extent of phosphorylation of 4EBP1 [90].

Despite these promising results, it is not yet known the synergistic mechanism of action between the anti-EGFR and mTOR inhibitors and, furthermore, the importance of EIF4EBP1 gene is not completely clear. This topic was recently treated by Madden et *al*. that, using gefitinib (anti-EGFR) and temsirolimus (anti-mTOR) on TNBC cell lines, discovered the presence of a cross-talk mechanism between EGFR and mTOR also engaging the eukaryotic translation initiation factor 4B (eIF4B) [91]. Moreover, the action of these two molecules would seem to block phosphorylation of eIF4B, finally resulting in a growth and survival reduction in TNBC cell lines and then suggesting to investigate mTOR inhibition in association with other drugs [92, 93].

Recently, Bhola et *al*. [70] have suggested that resistance to TORC1/2 inhibitors may be exceeded via inhibition of the FGFR-mitochondrial metabolism-Notch1 axis that allows to eradicate therapy-resistant cancer stem cells in TNBC.

**Table 1:** Ongoing trials studying the role of mTOR inhibitors in TNBC

TRIAL	REGISTRATION NUMBER	INVESTIGATOR INSTITUTION
Phase Ib/II Trials of RDA001 in Triple Negative Metastatic Breast Cancer	NCT01939418	National Cancer Center, Korea
A study of Lapatinib in combination with Everolimus in patients with Advanced, Triple Negative Breast Cancer	NCT01272141	Emory University Winship Cancer Institute
Liposomal Doxorubicin, Bevacizumab and Temsirolimus (DAT) in Triple-Negative Breast Cancer (TNBC) Insensitive to Standard Neoadjuvant chemotherapy	NCT02456857	M.D. Anderson Cancer Center
Comparison of Single-Agent Carboplatin vs the Combination of Carboplatin and Everolimus for the Treatment of Advanced Triple-Negative Breast Cancer	NCT02531932	Icahn School of Medicine at Mount Sinai
Eribulin Mesylate and Everolimus in Treating Patients With Triple-Negative Metastatic Breast Cancer	NCT02120469	City of Hope Medical Center
NECTAR Everolimus Plus Cisplatin (-) Breast Cancer (NECTAR)	NCT01931163	The Methodist Hospital System
Safety and Tolerability of Everolimus in Combination With Eribulin in Triple-negative Breast Cancer	NCT02616848	Istituti Ospitalieri di Cremona
A Study of AZD2014 in Combination With Selumetinib in Patients With Advanced Cancer (TORCMEK)	NCT02583542	Queen Mary University of London

## PI3K/AKT INHIBITORS

The PAM pathway may be targeted trough a different strategy involving the inhibition of its upstream targets such as PI3K and Akt [94]. While there are inhibitors inactivating both PI3K and mTOR, further development may be limited by issues, including increased toxicity [95]. Kalinski et *al*. have shown that subjecting patients affected by stage I/III BC (including 3 women TN) at different doses of MK-2206, an allosteric inhibitor of AKT, they experienced rash and pruritus G3, mucositis G2, fever G2 and hyperglycemia G2, leading to the trial suspension, despite two dose reductions [96].

A further setting in which the PI3K/AKT inhibitors could prove their usefulness could be in association to PARP inhibitors (PARPis) in TNBC patients who did not exhibited BRCA1/2 function loss [97]. This is because, as it is already known, PARPis result active in tumors deficient in the homologous recombination (HR) mechanisms due to alterations in the *BRCA1/2* genes [98-101], whereas their action is very negligible in non-BRCA mutant cancers [102].

Since the PI3K/AKT pathway stabilizes the function of HR, Ibrahim and collaborators have demonstrated that the use of AKT inhibitors in TNBC cell lines without BRCA1/2 alterations could cause HR function changes, and then sensitize to PARPis. In particular, the study showed that TNBC cancer cells treated with buparlisib (AKT inhibitor) were subject to a subsequent hyperactivation of ERK and MEK1, two essential component of the MAP kinase signal transduction pathway, resulting in downregulation of BRCA1 and then favoring the action of olaparib (PARPi) with subsequent reduction of cell proliferation and survival [103]. An interesting *in vitro* study showed that targeting multiple kinases such as IGF-1R, PI3K, mTORC or MEK may suppress cell proliferation and induce apoptosis in MDA-MB-231 cells, increasing also the inhibition of Akt phosphorylation [104].

A similar experience has been carried out by Kimbung et *al*. which evaluated the association between Rucaparib (PARPi) and LY294002 (PI3Ki) in BRCA1-deficient cells with the intent to improve the response to PARPis. This study showed promising results with sub-micromolar doses of both drugs, providing then a strong rationale for further research especially in TNBC [105].

The main clinical trials concerning the function of PI3K inhibitors in TNBC and currently under evaluation are reported in Table 2.

**Table 2:** Ongoing trials studying the role of PI3K inhibitors in TNBC

TRIAL	REGISTRATION NUMBER	INVESTIGATOR INSTITUTION
Capecitabine +BKM120 TNBC Brain Mer	NCT02000882	US Oncology Research
Phase I Study of the Oral PI3kinase Inhibitor BKM120 or BYL719 and the Oral PARP Inhibitor Olaparib in Patients With Recurrent Triple Negative Breast Cancer or High grade Serous Ovarian Cancer	NCT01623349	Dana-Farber Cancer Institute
Phosphatidylinositol 3-kinase (PI3K) Alpha iNhibition In Advanced Breast Cancer (PIKNIC)	NCT02506556	Peter MacCallum Cancer Centre, Australia

## CONCLUSIONS

TNBC is a heterogeneous subtype of BC showing aggressiveness and high risk of relapse [106].

In the last years, the treatment of metastatic breast cancer has seen the development of new systemic treatments. Despite this progress, TNBC still has limited therapeutic options: cytotoxic chemotherapy is the standard of care; systemic treatment tipically has transitory efficacy and the response is early followed by disease progression.

TNBC patients exhibit, indeed, an unfavorable outcome compared to those with other subtypes.

Only recently driver mutations have been identified with encouraging results in preclinical models and have allowed to investigate new specific drugs for each different subtype of the disease.

The PI3K-AKT-mTOR pathway is an exciting target for developing new anticancer therapeutics [107]. Since several pathways may be involved, therefore, the best results are achieved by combining different molecules on various targets paying attention to toxicity. For these reasons, it will be necessary in the near future to rescue pathological tissue taken before and after therapy in order to better understand the mechanisms of drug resistance and new concepts on tumor pathogenesis. Using increasingly refined techniques, liquid biopsies could have an important role, allowing us to obtain a lot of information in a short time and in a minimally invasive manner and maintaining a high concordance rate with the primary tumor and/or metastases. Therefore, is desirable enrolling TNBC patients with different subtypes in new specific trials to ensure them the most suitable treatment.

One of the main difficulties in the field of targeted therapies is represented by the extreme heterogeneity of the disease. This condition produces a number of different cases, each constituted by the presence of a rare mutation mostly detected in cases of exceptional responders in the context of negative trials. Consequently, although the discovery of these rare mutations is leading to the approval of many new antitumor therapies, it is necessary to design new studies showing benefits, avoiding all the problems related to the extreme heterogeneity of the disease contained in other conventional trials. A possible solution could be derived from the use of the so called basket trials that find their maximum indication where neoplasia depends on the pathway of the target and whether the therapy can effectively inhibit the action of the target itself. This would make possible to consider a marker as a probable predictor regardless of tumor histology.
